# Zinc(II) Iminopyridine Complexes as Antibacterial Agents: A Structure-to-Activity Study

**DOI:** 10.3390/ijms25074011

**Published:** 2024-04-04

**Authors:** Silvia de la Mata Moratilla, Sandra Casado Angulo, Natalia Gómez-Casanova, José Luis Copa-Patiño, Irene Heredero-Bermejo, Francisco Javier de la Mata, Sandra García-Gallego

**Affiliations:** 1 University of Alcalá, Faculty of Sciences, Department of Organic and Inorganic Chemistry and Research Institute in Chemistry “Andrés M. del Río” (IQAR), 28805 Alcalá de Henares, Spain; silvia.matam@edu.uah.es (S.d.l.M.M.); sandra.casado@edu.uah.es (S.C.A.); javier.delamata@uah.es (F.J.d.l.M.); 2 University of Alcalá, Faculty of Pharmacy, Department of Biomedicine and Biotechnology, 28805 Alcalá de Henares, Spain; natalia.gomezc@uah.es (N.G.-C.); josel.copa@uah.es (J.L.C.-P.); 3 Networking Research Center on Bioengineering, Biomaterials and Nanomedicine (CIBER-BBN), 28029 Madrid, Spain; 4 Institute Ramón y Cajal for Health Research (IRYCIS), 28034 Madrid, Spain

**Keywords:** Schiff base, iminopyridine, zinc, metal complex, bacteria, antibiotic, *Staphylococcus aureus*, *Escherichia coli*

## Abstract

Antibiotic resistance is currently a global health emergency. Metallodrugs, especially metal coordination complexes, comprise a broad variety of candidates to combat antibacterial infections. In this work, we designed a new family of Schiff base zinc(II) complexes with iminopyridine as an organic ligand and different inorganic ligands: chloride, nitrate, and acetate. The antibacterial effect of the Zn(II) complexes was studied against planktonic bacterial cells of *Staphylococcus aureus* (Gram-positive) and *Escherichia coli* (Gram-negative) strains. The results showed a moderate biocide activity in both types of planktonic bacteria, which arises from the metal complexation to the Schiff base ligand. Importantly, we confirmed the crucial effect of the metal, with Zn(II) improving the activity of Cu(II) counterparts previously reported. On the other hand, the impact of the inorganic ligands was not significant for the antibacterial effect but was relevant for the complex solubility. Finally, as proof of concept of topical antibacterial formulation, we formulated an emulsion containing the most lipophilic Zn(II) complex and confirmed a sustained release for 24 h in a vertical cell diffusion assay. The promising activity of iminopyridine Zn(II) complexes is potentially worth exploring in more detailed studies.

## 1. Introduction

Antibiotic resistance is a worldwide health emergency. Resistance to all antibiotics in clinical use has been detected, so next-generation antimicrobial therapies must be developed [1]. An encouraging approach is the use of metallodrugs [2]. These compounds have been employed for antibacterial treatment since ancient times. It is well known that first-row transition metals (manganese, iron, cobalt, nickel, copper, and zinc) are the key metals required by most organisms. Among them, zinc is unique since it possesses a filled d-orbital and does not undergo redox cycling. Furthermore, zinc is crucial in normal host immune function, as macrophages adopt different strategies of zinc starvation and zinc toxicity to kill the bacteria they phagocytize [3]. Inspired by this mechanism, different Zn metallodrugs have been designed, such as antibacterial ZnO nanoparticles [4].

Unlike solvated metal ions or metal nanoparticles, organometallic and metal coordination complexes present a well-defined arrangement of ligands around the metal center, thus expanding their versatility and applications [5,6]. The outstanding tunable properties of metal complexes arise from the vast variety of coordination numbers, geometries, structural diversity, and kinetics of ligand exchange. This structural versatility translates into a range of different mechanisms of action, such as redox reactions, ligand exchange processes, the generation of Reactive Oxygen Species (ROS), and the competitive inhibition of enzymes.

Schiff base ligands are a promising family of organic ligands in such metallodrugs [7]. The metal chelation to the Schiff base increases the lipophilicity of the ligand due to the delocalization of π-electrons in the entire chelate system, and thus increases the penetration through cell membranes. A broad list of Schiff base metal complexes mainly based on Cu(II), Ru(II), Ni(II), or Zn(II) show antibacterial activity [8,9]. It is important to mention that Zn(II) complexes frequently exhibit superior antimicrobial activity than other metals, like their Cu(II) counterparts. For example, a benzimidazole Schiff base Zn(II) complex exhibited an inhibition zone of 18.9 mm in *E. coli*, while for the Cu(II) analog, the zone was only 11.8 mm [10]. Another organometallic Schiff base Zn(II) complex produced inhibition zones of 26 mm and 17 mm against *E. coli* and *S. aureus*, respectively, while no activity was reported for the Cu(II) counterpart [11]. This promising activity, together with the biocompatibility and lower cost of zinc salts, enhances the potential of these metallodrugs.

Silane-containing Schiff base ligands further tune the lipophilic character and thus the biological activity of metal complexes. İspir et al. synthesized Zn(II) and Cd(II) complexes comprising 3-iminopropyltrimethoxysilane and 3-iminopropyltriethoxysilane chains, which exhibited certain antibacterial and antifungal activities [12]. In our previous works, we demonstrated the antibacterial activity of carbosilane iminopyridine complexes of Cu(II) and Ru(II) against *S. aureus* and *E. coli* [13]. All complexes exhibited better activity than the corresponding metal salts precursors, confirming the impact of the metal coordination to the iminopyridine ligand. Furthermore, relevant differences were found in the Minimum Inhibitory Concentration (MIC) and the Minimum Bactericide Concentration (MBC) depending on the type of metal or the counterions in the complex. Monometallic complexes were employed to predict the behavior of first- and second-generation carbosilane metallodendrimers, where the multivalent properties significantly improved the antibacterial effect [14]. This multivalency, characteristic of dendritic materials, is responsible for their outstanding properties for the prevention, treatment, and diagnosis of infectious diseases [15]. However, the combination of dendritic scaffolds with metal ions further expands the versatility and efficacy of the candidates by merging the activity of both components.

Considering the well-documented antibacterial activity of Schiff base Zn(II) complexes, in this study, we focused on the design of Zn(II) carbosilane iminopyridine complexes with three different inorganic ligands: chloride, nitrate, and acetate. These complexes exhibited moderate antibacterial activity against Gram-positive and Gram-negative bacteria and could be formulated as an emulsion for antibacterial topical uses. We confirmed the impact of the type of metal center and the ligands on the structure, as well as the antibacterial activity of these metallodrugs, finding that Zn(II) iminopyridine complexes appear as promising candidates for antibacterial therapy.

## 2. Results

### 2.1. Synthesis of Zn(II) Iminopyridine Complexes

**Figure 1:**
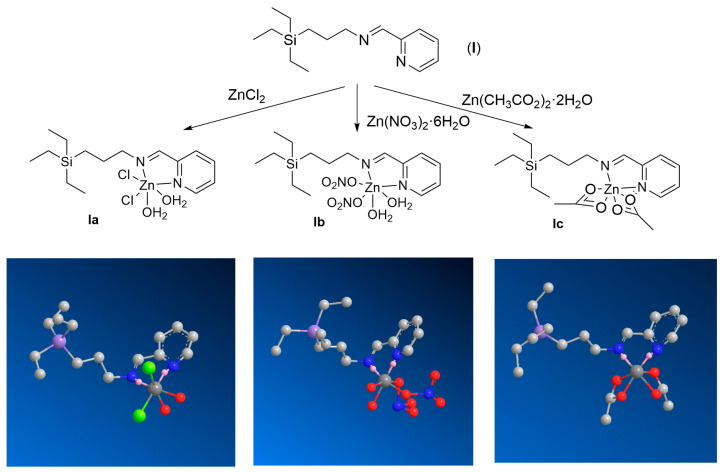
Synthetic protocol to prepare the Zn(II) iminopyridine complexes **Ia**–**c** and proposed structures. The reactions proceeded in MeOH for 12 h at room temperature, using 1:1 (M:L) stoichiometry. Insert: Snapshots from 3D spatial arrangement of complexes **Ia**–**c** in solid state after energy minimization. Hydrogen atoms have been hidden for simplicity. [Chem3D 22.0.0 software: job 1 (minimize energy to minimum RMS gradient of 0.010)].

The synthesis of the iminopyridine ligand (**I**) was carried out as described in [16]. This ligand was employed to synthesize three different Zn(II) complexes with several inorganic ligands: chloride, nitrate, and acetate (Figure 1). As Zn(II) precursors, ZnCl_2_, Zn(NO_3_)_2_·6H_2_O and Zn(CH_3_CO_2_)_2_·2H_2_O were used. In all the reactions, 1:1 stoichiometry (ligand:Zn) was employed. The ligand and the metal salt were dissolved in MeOH, mixed, and reacted for 12 h at room temperature. The complexes **Ia**, **Ib**, and **Ic** were isolated as brown oils with high yield after the evaporation of the solvent.

### 2.2. Structural Characterization of Zn(II) Complexes: Impact of the Metal Coordination and the Nature of the Inorganic Ligands

**Figure 2:**
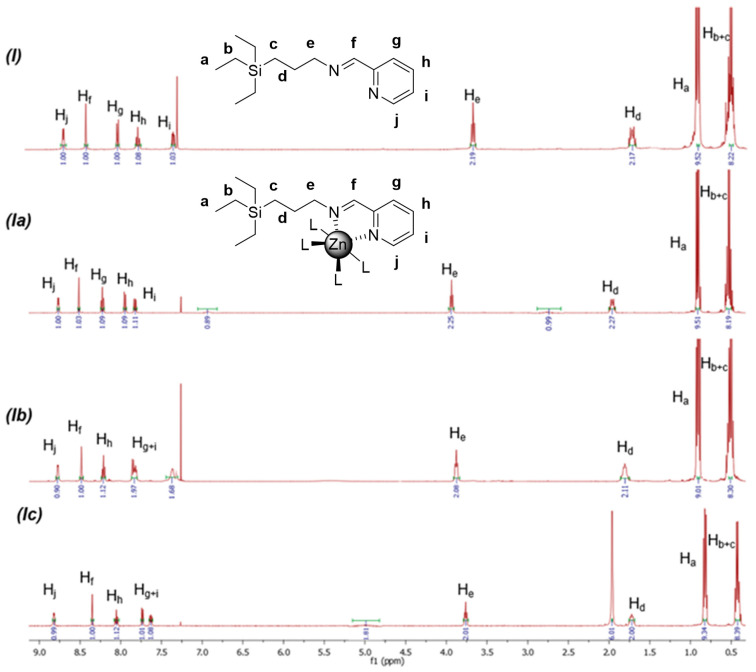
Comparative ^1^H-NMR spectra of compounds **I**, **Ia**–**c** in CDCl_3_.

To evaluate the impact of structural parameters, Zn(II) complexes **Ia**–**c** were comparatively studied through ^1^H-NMR and ^13^C-NMR (Figure 2 and Figure S1). Unlike the Cu(II) counterparts, the diamagnetic properties of Zn(II) complexes facilitated this study, which offered useful insight into the impact of the different structural parameters. The signals were compared to the precursor ligand **I**. In ^1^H-NMR spectra, it can be observed that all the signals close to the metal center appeared at lower field, compared to the precursor **I**. The signal corresponding to the methylene group connected to the N atom appeared at 3.66 ppm in the ligand, and it was shifted to 3.95 (**Ia**), 3.88 (**Ib**), and 3.76 (**Ic**). For the imine group, the signal was shifted from 8.37 (**I**) to 8.51 (**Ia**), 8.48 (**Ib**), and 8.34 (**Ic**). Additionally, relevant differences were observed in the shifting for the aromatic protons (Figure 2), probably as a result of the distortion of the aromatic ring through the chelation of the metal ion. The overall shifting can be explained by the removal of electronic density from the ligand, caused by the metal atom, as well as the chelating effect. A similar effect was observed in ^13^C-NMR (Figure S1). The imine carbon appeared in the range of 159.6–161.5 ppm for the new metal complexes.

Among the three Zn(II) complexes, relevant differences were also observed in NMR spectra, which can be ascribed to several reasons: The first reason is the different nature of the inorganic ligands. All of them are σ,π-donating ligands whose base strength decreases in the following order: CH_3_CO_2_^−^ > NO_3_^−^ > Cl^−^. As Zn(II) is a borderline Lewis acid, it will find higher affinity towards chloride ligands. The second reason is the different coordination modes of the ligands. All the complexes probably present the same coordination index (6) in solid state, which may vary in solution. However, the acetate ligands appear as bidentate ligands, as later confirmed through FTIR. This result is in line with the zeta potential values (Table 1). The Zn(II) complexes exhibited slightly negative values, and the negative charge decreases when increasing the metal–ligand affinity, reaching values close to zero for the chloride **Ia**. Again, Zn(II) complexes behave differently to the Cu(II) and Ru(II) counterparts previously reported, with clearly positive values. The fully filled d^10^ electronic configuration of Zn(II) liable with its redox inertness may be behind this behavior. Other Schiff base complexes of d^10^ metals (Zn(II), Cd(II) and Hg(II)) have been reported as antibacterial agents [17]. To predict the composition of our complexes, we compared to similar Zn(II) complexes bearing bidentate ligands with the iminopyridine moiety, such as 2-pyrilidineaniline [18] or 4-(4-aminophenoxy)-N-(1-(pyridin-2-yl)ethylidene)aniline [19]. In these examples, the authors confirmed a ZnLCl_2_(H_2_O)_2_ composition. However, in our complexes, the analysis of the ^1^H-NMR spectra in CDCl_3_ showed broad signals which, overall, integrated for around one water molecule in each complex. The exchange with the deuterated solvent might be responsible for this underestimation. Other analytical techniques were used to gain further insight into the composition of the complexes.

These complexes, in solid-state form, were explored through FT-IR (Figures S2–S4). The samples were dried under vacuum overnight to eliminate non-coordinated water and facilitate the interpretation of the spectra. The precursor ligand presented a characteristic band around 1650 cm^−1^, assigned to the imine C=N stretching. For the chloride complex **Ia**, the spectrum showed a shifting of this band to ~1600 cm^−1^. Additionally, a band in the 3400–3100 cm^−1^ range confirms the presence of water molecules. This corresponds to coordinated water, as two bands around 880–980 cm^−1^ can be observed due to ν(H_2_O) rocking and the wagging modes of vibrations [20]. For the nitrate complex **Ib**, the C=N band was also observed around 1650 cm^−1^. The characteristic stretching of monodentate nitrate ligands were observed at 1480, 1300, and 1010 cm^−1^, [21], and the presence of coordinated water molecules was observed in the ranges of 3500–3000 and 820–920 cm^−1^. Finally, for the acetate complex **Ic**, two broad peaks at 1400 and 1600 cm^−1^, assigned to the symmetric and asymmetric stretching of COO, were observed, which overlap with the peak from C=N stretching. In this case, no water molecules were observed. To explore the coordination mode of acetate, we evaluated the divergence of ν_asym_(COO) and ν_sym_(COO). The precursor Zn(acet)_2_·2H_2_O is described as octahedral, with two chelating acetate groups and two water molecules, and ∆ν = 145 cm^−1^ [22]. For **Ic**, we found a similar ∆ν, so we proposed a similar bidentate coordination of the ligands in the complex as a consequence of the elimination of the labile water molecules in the octahedral environment of the precursor that were replaced by the iminopyridine ligand. It has been described that ∆ν follows the following trend: chelating < bridging < ionic < monodentate [23]. In our case, sodium acetate exhibits ∆ν = 164 cm^−1^, so chelating coordination mode occurs when ∆ν << 164 cm^−1^, and bidentate bridging coordination occurs when ∆ν ≤ 164 cm^−1^. In complex **Ic**, the divergence ∆ν = 138 cm^−1^, so we can propose a chelating mode of the acetates. The Zn-O stretching appeared in the 500–570 cm^−1^ range, and the Zn-N stretching appeared in the 400–470 cm^−1^ range [24]. To further confirm the composition of our compounds, we performed an elemental analysis and ICP-OES. The hygroscopicity of our compounds was responsible for the slight deviations from the values calculated in elemental analysis. For example, for complex **Ic**, the experimental analysis matched the calculated values, considering the presence of one non-coordinated water molecule. This molecule was not observed in the FTIR analysis, when the sample was tested just directly after drying. ICP-OES revealed a higher content of Zn compared to the calculated values. Such deviations, with no clear explanation, highlighted the need for the optimization of the analysis parameters and sample preparation to be able to accurately quantify the amount of zinc in our complexes.

Additionally, we studied the stability of our complexes. We dissolved our complexes in water and analyzed the potential changes in the ^1^H-NMR spectra at t = 0 and t = 7 days. Samples were evaporated and redissolved in MeOD for analysis. After one week in water solution, we observed no changes in the ^1^H-NMR spectra (Figures S8–S10). These results are in agreement with former studies on the stability of Ru(II) complexes of the chelating ligand **I** [16]. The authors did not observe any appreciable changes in NMR spectra in D_2_O, PBS-D_2_O, or neat DMSO-d^6^, even after 72 h. However, similar monodentate complexes showed the displacement of the ligand from the metal coordination sphere at high DMSO ratios.

Furthermore, the thermal stability was explored through TGA (Figures S5–S7). The thermogravimetric study indicated that chloride complex **Ia** presented a small humidity loss around 85 °C and required temperatures around 250 °C to lose the two water molecules (8.3% weight loss), confirming their coordination to the central metal atom. For the nitrate complex **Ib**, the 7.4% loss, corresponding to two water molecules, was observed at above 125 °C, and the subsequent loss of the nitrate ligands was observed at above 190 °C. Again, this confirms the presence of coordinated water molecules in the coordination sphere of the central metal ion. These water molecules are more strongly bonded to the metal ions and, therefore, eliminated at higher temperatures. For the acetate complex **Ic**, the loss of non-coordinated water occurred at around 85 °C, and that for the acetate ligands occurred at above 190 °C. Overall, we observed a higher thermal stability for the chloride derivative compared to the nitrate and acetate counterparts. However, the three complexes were stable under the conditions required for the biological evaluation.

Other similar ligands, but with a tridentate NNO nature, like 2-[[(2-pyridinylmethyl)imino]methyl]phenol (HSALIMP), generated two types of complexes: the dimeric [Zn(SALIMP)NO_3_]_2_ and the monomeric [Zn(HSALIMP)Cl_2_]·H_2_O [25]. In these examples, the ligand acted as a tridentate in the presence of the moderately coordinating nitrate or as a bidentate in the presence of the anionic chloride. In order to evaluate the nuclearity of our complexes, we performed High-Resolution Mass Spectrometry (Figure S11). Unfortunately, we could not identify the molecular peak of any of our complexes. However, the results pointed to mononuclear species, and only in the case of complex **Ic** may the presence of small peaks above m/z 700 indicate the presence of minor binuclear species. These binuclear species could be explained by bridging acetate ligands, but only as minor species, according to the FTIR spectra.

### 2.3. Antibacterial Activity

#### 2.3.1. Biocidal Activity

The tested compounds showed antibacterial activity against *S. aureus* and *E. coli*. The MIC values of the compounds were 64 mg/L for *S. aureus* and 128 mg/L for *E. coli*, except for **Ic**, with a range of 64–128 mg/L (Table 1). This confirmed that they are bactericidal compounds whose MBC values correspond with the MIC values. The biocidal activity was compared with the precursor ligand **I** as well as with the precursor salts (ZnCl_2_, Zn(NO_3_)_2_·6H_2_O, Zn(CH_3_CO_2_)_2_·2H_2_O), with all of them exhibiting MIC and MBC values higher than 512 mg/L.

#### 2.3.2. Kinetic Studies

Kinetic studies showed that bacteria treated with compounds **Ib** and **Ic** required 2 h more than the control to grow for the *S. aureus* strain and 1 h for the *E. coli* strain at one concentration below their MIC values. However, the chloride complex **Ia** inhibited the growth of both bacteria up to 3 h later than the control at one concentration below the MIC value. This confirmed the impact of the type of inorganic ligand. In the studies against *E. coli*, the bacterial population treated with this compound was unable to reach a viability of 50% at a concentration below its MIC (64 mg/L) at 20 h, while in *S. aureus*, a viability of 90.9% was recorded at a concentration below its MIC (32 mg/L) at the same time.

### 2.4. Evaluation of Topical Antibacterial Emulsion

According to the zeta potential values, the chloride complex **Ia** exhibited more lipophilic properties. This could be interesting for topical antibacterial action, where the complex must cross the skin barriers. Furthermore, kinetic studies revealed a prolonged inhibitory effect of the chloride derivative compared to the nitrate and acetate complexes. As proof of concept, compound **Ia** was formulated in a W/O emulsion (at 4%) comprising glycerin, milliQ water, PEG-400, and Emolivan^TM^ base (see the Section 4 Materials and Methods). A thorough quality check confirmed the correct formation of the emulsion.

**Figure 3:**
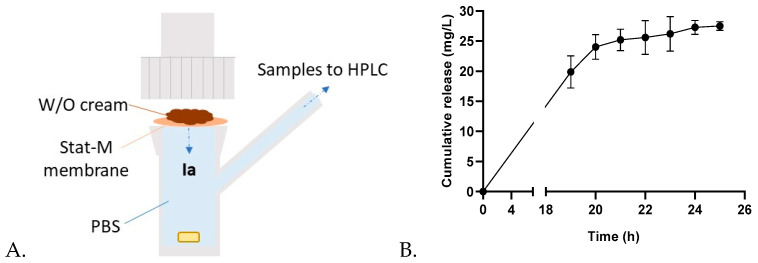
(**A**) Scheme depicting the vertical cell for the diffusion assay. (**B**) Cumulative release of compound **Ia** from a 4% W/O cream formulation at 32 °C. In the donor chamber, 55 mg of the cream was applied on the membrane. The collector chamber was filled with PBS. Average results from two independent experiments are represented in the graph.

Subsequently, the release of **Ia** from the formulation was studied through a vertical diffusion cell HDT-1000 using Stat-M membranes at 32 °C to simulate skin conditions (Figure 3). The emulsion (total 55 mg, containing 2.2 mg of **Ia**) was deposited on the membrane in the donor chamber, and PBS was used in the collecting chamber. Aliquots of 0.2 mL were collected at different times, filtered through a 0.2 µm filter, and evaluated through HPLC. As represented in Figure 3B, the drug release reached a plateau after 24 h, which matches the loss of the brownish color in the emulsion. This assay confirmed the potential of Zn(II) iminopyridine complexes as antibacterial candidates in topical formulations, enabling a sustained release.

## 3. Discussion

Schiff base metal complexes are promising therapeutic metallodrugs with applications in different fields, such as cancer therapy, or they could be used as antimicrobial agents [7,8,9]. In our previous studies, we explored the activity of Schiff base complexes comprising a carbosilane iminopyridine ligand, as well as different metal ions such as Cu(II) and Ru(II) [13]. These complexes exhibited promising antitumor and antimicrobial activity and, more importantly, could emulate the behavior of their multivalent counterparts: metallodendrimers. The monometallic complexes enabled a simplified evaluation of the impact of different structural parameters, like the type of metal ion and the nature of the inorganic ligands. We concluded that these parameters are crucial for the biological activity of these metallodrugs.

In line with our former studies, in this work we designed a family of Zn(II) iminopyridine-containing metallodrugs and evaluated the influence of these structural parameters on their antimicrobial activity (Figure 1). These complexes were prepared through a straightforward reaction and isolated with high yields. Their structural characterization through NMR, FTIR, elemental analysis, ICP, TGA, and HRMS offered relevant information about the purity, structure, and stability of the complexes. The new Zn(II) compounds probably exhibit an octahedral coordination in solid state and are stable in water solution for long periods of time and at temperatures below 120 °C. As expected, the metal ion coordination to the iminopyridine ligand induced a shift in the ^1^H and ^13^C NMR signals, potentially explained by the “heavy atom-light atom” (HALA) effect [26]. For example, Barone et al. explored the effect of different metal ions (Ni(II), Pd(II), Pt(II), and Zn(II)) on the ^1^H-NMR shift of Schiff base complexes [27]. They observed that the nearest protons to the metal center are the most deshielded, and the covalent nature of the M-O and M-N bonds significantly affected such deshielding. In our case, we observed a deshielding of H_e_ and H_f_ in the trend **Ia** > **Ib** > **Ic**, even reaching a small shielding of H_f_(**Ic**). We might interpretate this result as indicative of the more covalent nature of the Zn-N bond for the chloride complex, followed by the nitrate and the acetate systems.

Unexpectedly, the Zn(II) complexes exhibited slightly negative Z-potential values, and their charge increased in the following order: **Ia** < **Ib** < **Ic** (Table 1). This indicated that the most colloidally unstable and lipophilic compound was the chloride derivative, also confirmed by the strong interaction with the chloride and water ligands. Compared to the Cu(II) and Ru(II) counterparts, which showed strong cationic properties that could disrupt bacterial membrane through electrostatic interactions, the new Zn(II) derivatives seem to behave differently. A redox mechanism is also ruled out due to the d^10^ electronic configuration of zinc.

Similar Zn(II) Schiff base complexes have been described in the literature, with these ones mainly being used as catalysts in esterification reactions. Di Serio et al. modulated the Lewis acidity of zinc by altering the inorganic ligands in the iminopyridine complexes with different coordination abilities: Cl^−^ > AcO^−^ > CF_3_CO_2_^−^ > CF_3_SO_3_^−^ [28]. Through NMR and conductivity measurements, they confirmed that chloride and acetate were non-conductive, while the rest were readily displaced by solvent molecules affording cationic species. Our complexes exhibited a similar behavior, as shown through DLS. The same authors described a complex analogous to **Ic**, bearing a triethoxosilyl ligand instead of a triethylsilyl [29]. In these two cases, the authors depicted a tetrahedral environment around the Zn atom, but few characterization details were provided. Sharma et al. bound zinc acetate to silica-supported magnetic nanoparticles decorated with a similar ligand [30]. The C=N peak shifted from 1647 to 1636 cm^−1^ after metalation, but no studies were carried out on the coordination sphere of the metal atom. A more detailed characterization was found for a Zn(II) bis(imino)pyridine complex, where single-crystal X-ray diffraction confirmed the monomeric nature and trigonal bipyramidal geometry around the zinc center, with two monodentate acetate ligands [31]. The monodentate disposition of the acetate ligands is favored due to the presence of the tridentate Schiff base ligand, unlike the complex herein described.

The antibacterial effect of the Zn(II) complexes was evaluated by measuring the inhibitory (MIC) and bactericide (MBC) properties against Gram-positive and Gram-negative bacteria. From the results summarized in Table 1, we can draw the following conclusions:

(1) The metal complexation to the ligand, which was clearly demonstrated through the shifting of the signals in NMR and FTIR, is crucial for the antibacterial effect. The precursor ligand and the metal salts used as controls are inactive, but the chelating effect of the iminopyridine ligand stabilizes the metallodrug and increases its lipophilicity. Furthermore, it rules out the possibility that the antibacterial activity is related to metal release, as we previously demonstrated with similar complexes [13].

(2) Carbosilane metallodrugs are promising broad-spectrum antibiotics exhibiting activity against both *S. aureus* (Gram-positive) and *E. coli* (Gram-negative) bacteria. As expected, all compounds were more active against *S. aureus* than against *E. coli*. This effect is frequent because Gram-negative microorganisms are slightly more resistant to treatments than Gram-positive ones due to the outer membrane on the cell wall of Gram-negative bacteria [32].

(3) The nature of the inorganic ligand (chloride, nitrate, or acetate) produces a different shifting in the NMR signals, arising from the electronic withdrawal and the chelating effect. This is relevant for the solubility of the complexes (with the chloride system being the most lipophilic) but not significant for the antibacterial effect in these Zn(II) complexes. Similar MIC and MBC values were obtained for **Ia–c**, and just a slightly better activity was observed for the chloride complex **Ia** in the kinetic studies.

(4) The water solubility of complexes **Ia–c** is a relevant advantage for antibacterial applications. A great number of Schiff base metal complexes described in the literature exhibit very poor or no solubility in water; thus, the testing of their antibacterial activity through the inhibition zone assay is required. For example, Naureen et al. studied two different Zn(II) Schiff base complexes bearing monodentate acetate ligands, which exhibited 11 and 19 mm inhibition zones for *E. coli* and 11 and 15 mm inhibition zones for *S. aureus* [33]. Other examples are the Zn(II) and Cu(II) complexes from the ligand (E)-2-(((7-chloro-2-hydroxyquinolin-3-yl) methylene) amino)-3-phenylpropanoic acid [23]. At 100 µg/mL, they showed diameters of 21.08 and 6.08 (in *E. coli*) and 25.05 and 6.58 mm (in *S. aureus*), respectively. The different techniques used for the evaluation of the antibacterial activity hinders the accurate comparison of complexes **Ia–c** with others described in the literature. However, they can be compared to the Cu(II) and Ru(II) complexes bearing the same ligand [13].

(5) The type of metal ion is crucial for the antimicrobial activity. The chloride Zn(II) complex **Ia** and its Cu(II) counterpart exhibited similar activity in *S. aureus*, but the Zn(II) complex was slightly more active against *E. coli*. The nitrate Zn(II) complex **Ib**, though, improves the activity against both bacteria compared to its Cu(II) counterpart. This behavior is in line with former studies that confirm the higher antibacterial activity of Zn(II) Schiff base complexes compared to Cu(II) compounds [13]. Overall, we could establish the following trend regarding the antibacterial activity of carbosilane iminopyridine systems: Ru(II) complexes > Zn(II) complexes > Cu(II) complexes.

(6) The Z-potential values observed with these complexes rule out a main antibacterial mechanism due to the cationic charge. As we previously reported, the antibacterial properties must rely on other mechanisms related to the presence of metal ions. Zinc is a redox-inactive metal required for catalytic activity and/or structural stability for thousands of proteins. Bacterial pathogens have developed mechanisms to keep zinc homeostasis, relying on a complex network of metal transporters and zinc buffering systems [3]. For example, to overcome Zn intoxication, *E. coli* possesses a metalloregulator named ZntR, which causes DNA conformational changes, leading to the expression of the P-type ATPase ZntA. This mechanism, together with the expression of cation diffusion family transporters and the use of glutathione as a buffering agent, alleviates Zn toxicity. For *S. aureus*, bacteria produce the metallophore staphylopine with broad metal-chelating abilities, which regulates Zn homeostasis. The use of the metallodrugs designed in this study can affect Zn homeostasis, potentially explaining their antibacterial activity.

As proof of concept, we formulated the most lipophilic complex **Ia** as an emulsion and evaluated the topical diffusion behavior using membranes that simulated skin. The results indicated a complete release of the compound in the first 24 h, confirming the potential use of these metallodrugs in topical antimicrobial formulations.

Overall, we concluded that the promising results obtained with the Zn(II) complexes encourage us to design multivalent platforms such as carbosilane metallodendrimers to significantly improve the antimicrobial activity of the monometallic complexes and to understand the impact of the presence of several Zn(II) complexes in a single scaffold.

## 4. Materials and Methods

### 4.1. Synthesis and Characterization of Zn(II) Iminopyridine Complexes

Solvents were purified from appropriate drying agents. Chemicals were purchased from commercial sources and used as received. Elemental analyses were performed on a LECO CHNS-932. NMR spectra were recorded on a Varian Unity VXR-300 Hz and 500 Hz instruments (Varian, Inc., Palo Alto, CA, USA) in CDCl_3_. Chemical shifts are given in ppm. ^1^H and ^13^C resonances were measured relative to internal deuterated solvents peaks. {^1^H-^13^C}-HSQC-2D-NMR experiments were carried out to support the assignment of the signals. FT-IR spectra were recorded using a Cary 630 FTIR Agilent instrument (Agilent, Santa Clara, CA, USA) equipped with an attenuated total reflectance (ATR) accessory. Agilent Microlab PC software v5.1 was used for spectra acquisition, which was carried out from 4000 to 400 cm^−1^. ICP studies were performed in an ICP-OES 720 Agilent (Agilent, Santa Clara, CA, USA) automatic injector SPS3 at λ 202.548 and 213.857 nm. The samples were digested in HNO_3_ (6 mL, ultrapure ppb trace quality, Scharlab, Barcelona, Spain) in a microwave digestor Milestone Ethos Easy (Sorisole, BG, Italy) at 230 °C for 20 min and then diluted to 25 mL with milliQ water. The calibration curve contained 0, 400, 1000, and 2000 ppb of Zn. TGA evaluation was performed in a TGA55 (TA Instruments, New Castle, DE, USA) in the range of 25–270 °C, with an increase of 10 °C/min. Spectra were analyzed with software Trios V5.6.0.87 (TA Instruments, New Castle, DE, USA). High-Resolution Mass Spectrometry studies were performed in a high-resolution Triple TOF 5600 plus (Sciex, Framingham, MA, USA).

General synthetic protocol: A MeOH solution of the metal salt (1 eq., in 4 mL) was added over a MeOH of the ligand **I** (1 eq., in 4 mL). After 24 h stirring at r.t., the solvent was evaporated, and the product was isolated as dark brown oil with high yield.

#### 4.1.1. G0[NCPh(o-N)ZnCl_2_·2H_2_O] (**Ia**)

**Ia** was prepared according to the general protocol using the following reagents: ligand **I** (1.27 mmol) and ZnCl_2_ (1.27 mmol). Yield: 90%.
^1^H-NMR: δ 8.42 (N=CH); 8.81, 8.20, 7.84 (4H, CH^py^); 3.95 (t, 2H, -C*H*_2_N); 1.98 (m, 2H, SiCH_2_C*H*_2_CH_2_N); 0.92 (m, 9H, SiCH_2_C*H*_3_); 0.54 (m, 8H, SiC*H*_2_CH_3_ & SiC*H*_2_CH_2_CH_2_N). ^13^C-NMR {^1^H}: δ 161.5 (N=*C*H); 146.5 (C^py^); 149.4, 142.1, 129.4, 128.2 (*C*H^py^); 61.8 (SiCH_2_CH_2_*C*H_2_N); 24.7 (SiCH_2_*C*H_2_CH_2_N); 8.9 (Si*C*H_2_CH_2_CH_2_N); 7.3 (SiCH_2_*C*H_3_), 2.7 (Si*C*H_2_CH_3_). Elemental analysis (%): C_15_H_30_Cl_2_N_2_O_2_SiZn (434.8 g/mol) Calc.: C, 41.44%; H, 6.96%; N, 6.44%. Exp.: C, 41.77%; H, 6.40%; N, 6.55%. FT-IR: v(C=N): 1643.8 cm^−1^. ICP (Zn%): Calc. 15.0%, Exp. 19.1%.

#### 4.1.2. G0[NCPh(o-N)Zn(NO_3_)_2_·2H_2_O] (**Ib**)

**Ib** was prepared according to the general protocol using the following reagents: ligand **I** (0.487 mmol) and Zn(NO_3_)_2_·6H_2_O (0.487 mmol). Yield: 93%.
^1^H-NMR: δ 8.35 (N=C*H*); 8.79, 8.19, 7.80 (4H, CH^py^); 3.88 (t, 2H, -C*H*_2_N); 1.81 (m, 2H, SiCH_2_C*H*_2_CH_2_N); 0.92 (m, 9H, SiCH_2_C*H*_3_); 0.54 (m, 8H, SiC*H*_2_CH_3_ & SiC*H*_2_CH_2_CH_2_N). ^13^C-NMR {^1^H}: δ 161.3 (N=*C*H); 146.1 (C^py^); 149.1, 141.1, 129.0, 127.4 (*C*H^py^); 61.3 (SiCH_2_CH_2_*C*H_2_N); 24.0 (SiCH_2_*C*H_2_CH_2_N); 8.5 (Si*C*H_2_CH_2_CH_2_N); 7.1 (SiCH_2_*C*H_3_), 2.8 (Si*C*H_2_CH_3_). Elemental analysis (%): C_15_H_30_N_4_O_8_SiZn (487.9 g/mol). Calc.: C, 36.93%; H, 6.20%; N, 11.48%. Exp.: C, 36.51%; H, 6.11%; N, 10.96%. FT-IR: v(C=N): 1645.6 cm^−1^. ICP (Zn%): Calc. 13.4%, Exp. 14.1%.

#### 4.1.3. G0[NCPh(o-N)Zn(OOCCH_3_)_2_] (**Ic**)

**Ic** was prepared according to the general protocol using the following reagents: ligand **I** (0.497 mmol) and Zn(O_2_CCH_3_)_2_·2H_2_O (0.497 mmol). Yield: 98%.
^1^H-NMR: δ 8.34 (N=C*H*); 8.82, 8.05, 7.73, 7.63 (4H, CH^py^); 3.76 (t, 2H, -C*H_2_*N); 1.91 (6H, C*H_3_*CO_2_); 1.73 (m, 2H, SiCH_2_C*H_2_*CH_2_N); 0.88 (m, 9H, SiCH_2_C*H_3_*); 0.40 (m, 8H, SiC*H_2_*CH_3_ & SiC*H_2_*CH_2_CH_2_N). ^13^C-NMR {^1^H}: δ 179.8 (CH_3_*C*O_2_); 159.6 (N=*C*H); 146.6 (C^py^); 149.9, 140.8, 128.4, 126.6 (*C*H^py^); 61.8 (SiCH_2_CH_2_*C*H_2_N); 24.2 (SiCH_2_*C*H_2_CH_2_N); 22.5 (*C*H_3_CO_2_); 8.9 (Si*C*H_2_CH_2_CH_2_N); 7.2 (SiCH_2_*C*H_3_); 3.0 (Si*C*H_2_CH_3_). Elemental analysis (%): C_19_H_32_N_2_O_4_SiZn (445.9 g/mol). Calc.: C, 51.17%; H, 7.23%; N, 6.28%. Calc.(+H_2_O): C, 49.19%; H, 7.39%; N, 6.04%. Exp.: C, 49.17%; H, 7.27%; N, 5.92%. Calc.: FT-IR: v(C=N) & v(C=O): 1593.4 cm^−1^. ICP (Zn%): Calc. 14.1%, Exp. 20.0%.

### 4.2. Formulation in W/O Emulssion

The cream was formulated using the following components: active principle (**Ia**) (40 mg), glycerin (50 mg), milliQ water (50 mg), polyethylene(glycol)-400 (60 mg), and Emolivan base (800 mg). In a flask, **Ia**, glycerin, PEG-400, and water were mixed. In another flask, Emolivan base was added and heated to 85 °C until fusion. The first mixture is heated to 85 °C and then added drop-wise to the Emolivan base while stirring with a glass pipette. The mixture was stirred until it reached room temperature. Subsequently, quality control was performed. The organoleptic control indicated that a viscous brown emulsion had been obtained. From the external/internal phase control, the test with Sudan III and methylene blue confirmed the formation of a W/O emulsion.

### 4.3. Vertical Cell Diffusion Assay

The assays were conducted in a HDT 1000 vertical diffusion cell tester from Copley Scientific using a vertical Franz skin cell with a nominal volume of the acceptor compartment of 12 mL and a diameter of 15 mm. For the donor compartment, the **Ia**-loaded emulsion was initially set. The experiments were conducted in triplicate and carried out at 37 °C and 480 rpm for 24 h. Samples were taken at different time points and quantified through HPLC. The extracted volume was continuously replaced to keep the volume constant. The assay was performed in aqueous media as the acceptor phase, mimicking physiological conditions corresponding to buffer solution (PBS) at pH 7.4. A transdermal diffusion test model Strat-M^®^ membrane was used. These membranes have two different layers, simulating human epidermis and dermis; thus, the permeation rates are slower than with other types of membranes.

Drug (compound **Ia**) release was quantified using an HPLC Agilent 1200 Series (Agilent, Santa Clara, CA, USA) with a DAD detector at 258 nm using water/acetonitrile (9:1) as the mobile phase. The calibration curve was performed with increasing amounts of the active principle **Ia**. Additionally, a blank emulsion was prepared without the active principle and evaluated through HPLC after dissolution in PBS.

### 4.4. Zeta Potential Evaluation

Zeta potential was measured using a Photon Correlation spectrometer Zetasizer Nano ZS (Malvern Instruments, Malvern, UK). The Helmholtz–Smoluchowski equation was used to calculate the final value. Five measurements in seven cycles of each sample were made. Compounds were measured in distilled water at a concentration of 30 µM. The data were analyzed using Zetasizer Software (version 7.11, Malvern Instruments Ltd., Malvern, UK).

### 4.5. Bacterial Strains

The bacterial strains used in this study were *Escherichia coli* (CECT 515, Gram-negative) and *Staphylococcus aureus* (CECT 240, Gram-positive), provided by the Spanish Type Culture Collection (CECT) in lyophilized form. The strains were kept at −20 °C and grown on Plate Count Agar (PCA).

### 4.6. In Vitro Antibacterial Activity Tests

The assay was based on the ISO 20776-1:2006 protocol [34]. First, colonies were transferred and grown in Mueller Hinton Broth medium (Scharlab S.L., Spain) and subjected to agitation (150 rpm) at 37 °C for 24 h. Then, the bacteria were incubated with the three compounds. The range of concentrations tested was from 0.5 to 512 mg/L. The three compounds were dissolved in water. Assays were carried out in sterile 96-well plates. Different controls were added, such as inoculum without compound, compound without inoculum, and culture medium without neither inoculum nor compound. Assays were run on technical triplicates. The treated plates were incubated at 37 °C for 24 h. Absorbance was measured at 630 nm in an Ultra Microplate reader (BIO-TEK Instruments, model ELx808, Winooski, VT, USA) after the incubation period. The results were collected to obtain the Minimum Inhibitory Concentration (MIC) values. Finally, well content was resuspended, and 5 µL of each well was deposited onto a PCA Petri dish and incubated at 37 °C for 24 h to obtain the Minimum Bactericidal Concentration (MBC) values.

### 4.7. Kinetic Studies

*S. aureus* and *E. coli* were grown as described in Section 4.6. Kinetic studies were performed by measuring absorbance values at 630 nm every hour up to 20 h. Microplates were incubated at 37 °C. All assays were performed in triplicate. The data at each time-point were analyzed by comparing the values of the different concentration gradients with the value of the control at the corresponding time. The cell viability percentage was calculated for each compound concentration and incubation time.

## Figures and Tables

**Table 1:** Minimum Inhibitory Concentration (MIC) and Minimum Bactericidal Concentration (MBC) effect of Schiff base complexes in planktonic cells and comparative values of Z-potential.

Compound	Zeta Potential, [mV]	*S. aureus* MIC [mg/L]	MBC [mg/L]	*E. coli* MIC [mg/L]	MBC [mg/L]
G0[NCPh(*o*-N)ZnCl_2_·2H_2_O] (**Ia**)	−0.33 ± 0.06	64	64	128	128
G0[NCPh(*o*-N)Zn(NO_3_)_2_·2H_2_O] (**Ib**)	−6.28 ± 0.33	64	64	128	128
G0[NCPh(*o*-N)Zn(O_2_CCH_3_)_2_] (**Ic**)	−8.43 ± 1.53	64	64	64–128	64–128
G0[NCPh(*o*-N)CuCl_2_·H_2_O] *^a^*	10.45 ± 1.25	64	64	256	256
G0[NCPh(*o*-N)Cu(NO_3_)_2_·H_2_O] *^a^*	14.79 ± 1.92	128	128	128	256
G0[NCPh(*o*-N)Ru(Cp)(PTA)]Cl *^a^*	18.70 ± 4.21	16	16	64	64

*^a^* previously published results [13].

## Data Availability

Data are contained within the article or Supplementary Materials.

## Supplementary Materials

The following supporting information can be downloaded at: https://www.mdpi.com/article/10.3390/ijms25074011/s1.
