# Supplementary material for: Zinc(II) Iminopyridine Complexes as Antibacterial Agents: A Structure-to-Activity Study
Source: Int J Mol Sci. 2024 Apr 4;25(7):4011. doi: 10.3390/ijms25074011 (PMC11012978; doi:10.3390/ijms25074011)
Supplement: Supplementary file 1 [file ijms-25-04011-s001.zip › ijms-2777788-supplementary.pdf]

Submitted to International Journal of Molecular Sciences

Article type: regular article

SUPPLEMENTARY INFORMATION

Zinc(II) Iminopyridine Complexes as Antibacterial Agents: A Structure-to-Activity Study

Silvia de la Mata Moratilla [1], Sandra Casado Angulo [1], Natalia Gómez-Casanova [2], José Luis Copa-Patiño [2], Irene Heredero-Bermejo [2,*], Francisco Javier de la Mata [1,3,4] and Sandra García-Gallego [1,3,4,*]

[1] University of Alcalá, Faculty of Sciences, Department of Organic and Inorganic Chemistry and Research Institute in Chemistry "Andrés M. del Río" (IQAR), 28805 Alcalá de Henares, Spain; silvia.matam@edu.uah.es (S.d.l.M.M.); sandra.casado@edu.uah.es (S.C.A.); javier.delamata@uah.es (F.J.d.l.M.)

[2] University of Alcalá, Faculty of Pharmacy, Department of Biomedicine and Biotechnology, 28805 Alcalá de Henares, Spain; natalia.gomezc@uah.es (N.G.-C.); josel.copa@uah.es (J.L.C.-P.)

[3] Networking Research Center on Bioengineering, Biomaterials and Nanomedicine (CIBER-BBN), 28029 Madrid, Spain

[4] Institute Ramón y Cajal for Health Research (IRYCIS), 28034 Madrid, Spain

* Correspondence: irene.heredero@uah.es (I.H.-B.); sandra.garciagallego@uah.es (S.G.-G.)

Table of Contents

Figure S1. ^{13}C-NMR spectra of compounds **Ia**, **Ib** and **Ic** in $CDCl_3$…………………………………………………..2

Figures S2-4. FTIR of compounds **Ia**, **Ib** and **Ic**, and precursors……………………..……………………………..3

Figures S5-7. TGA of compounds **Ia**, **Ib**, **Ic** and **I**….…………….…………………………….……….……….6

Figures S8-10. Stability study in water of compounds **Ia**, **Ib** and **Ic** at t=0 and t=7 days…………………...……7

Figure S11. HRMS study of compounds **Ia**, **Ib** and **Ic**…………………………………………………………..…..9

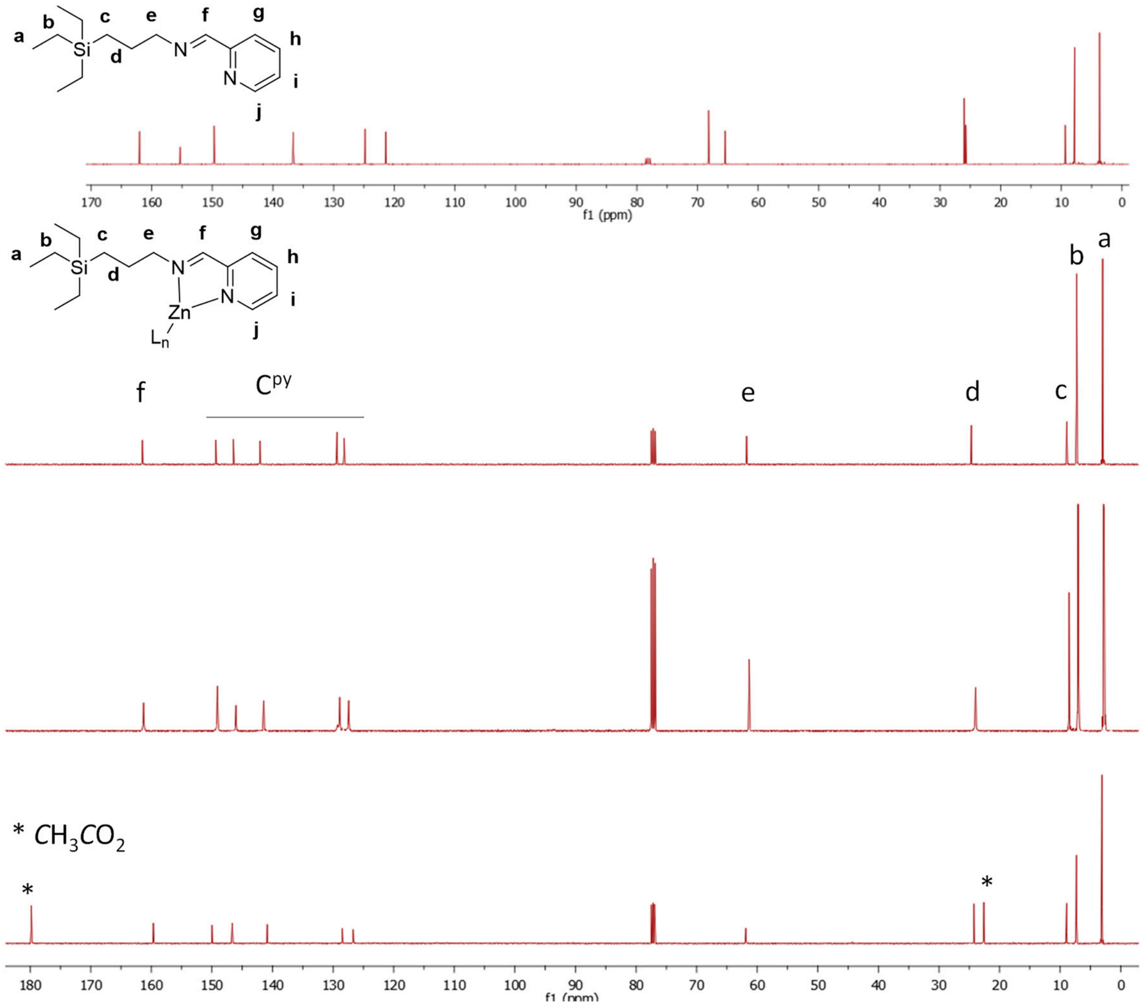

Figure S1. ^{13}C-NMR spectra of compounds **I**, **Ia**, **Ib** and **Ic** (from top to bottom) in $CDCl_3$.

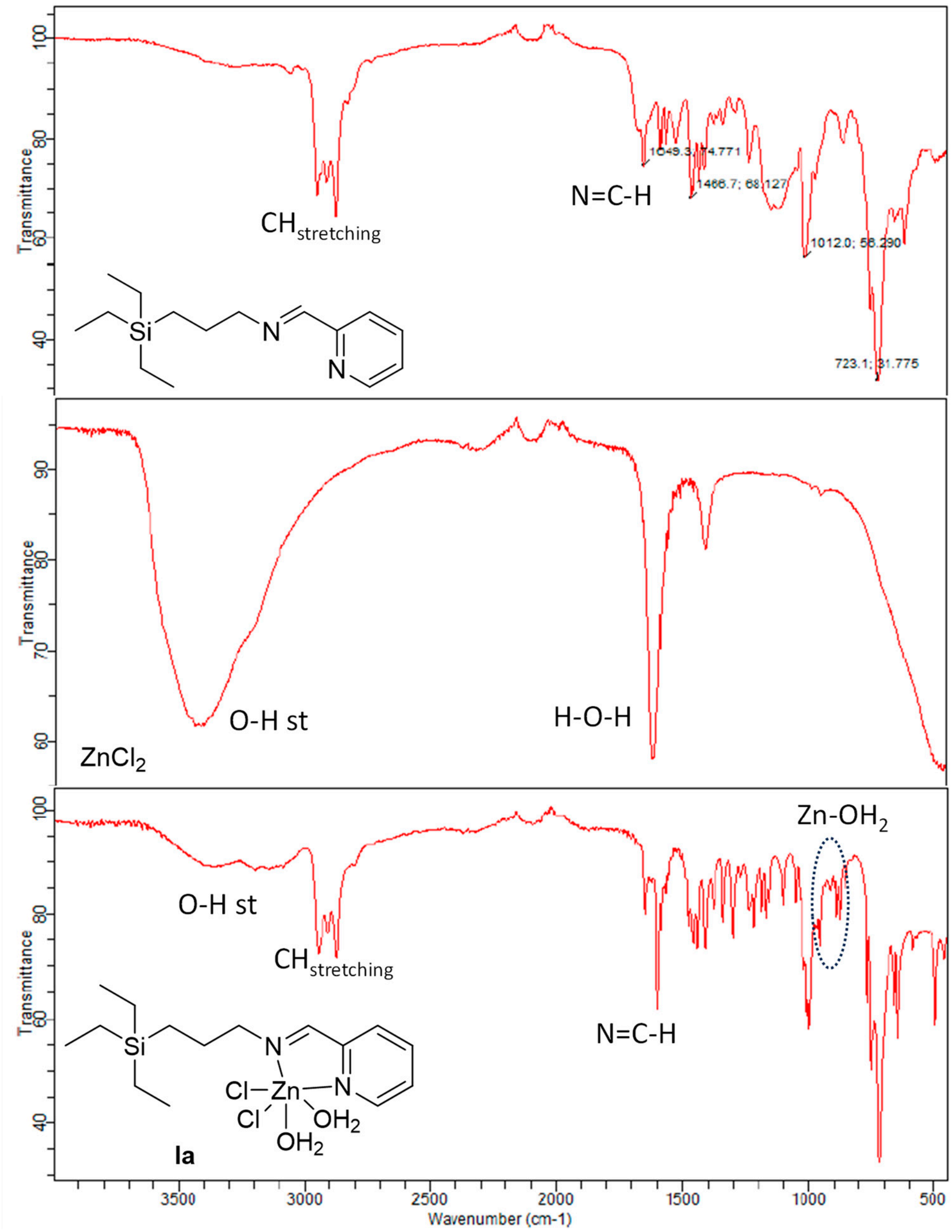

Figure S2. FTIR of compound **I** (top), $ZnCl_2$ (middle) and **Ia** (bottom).

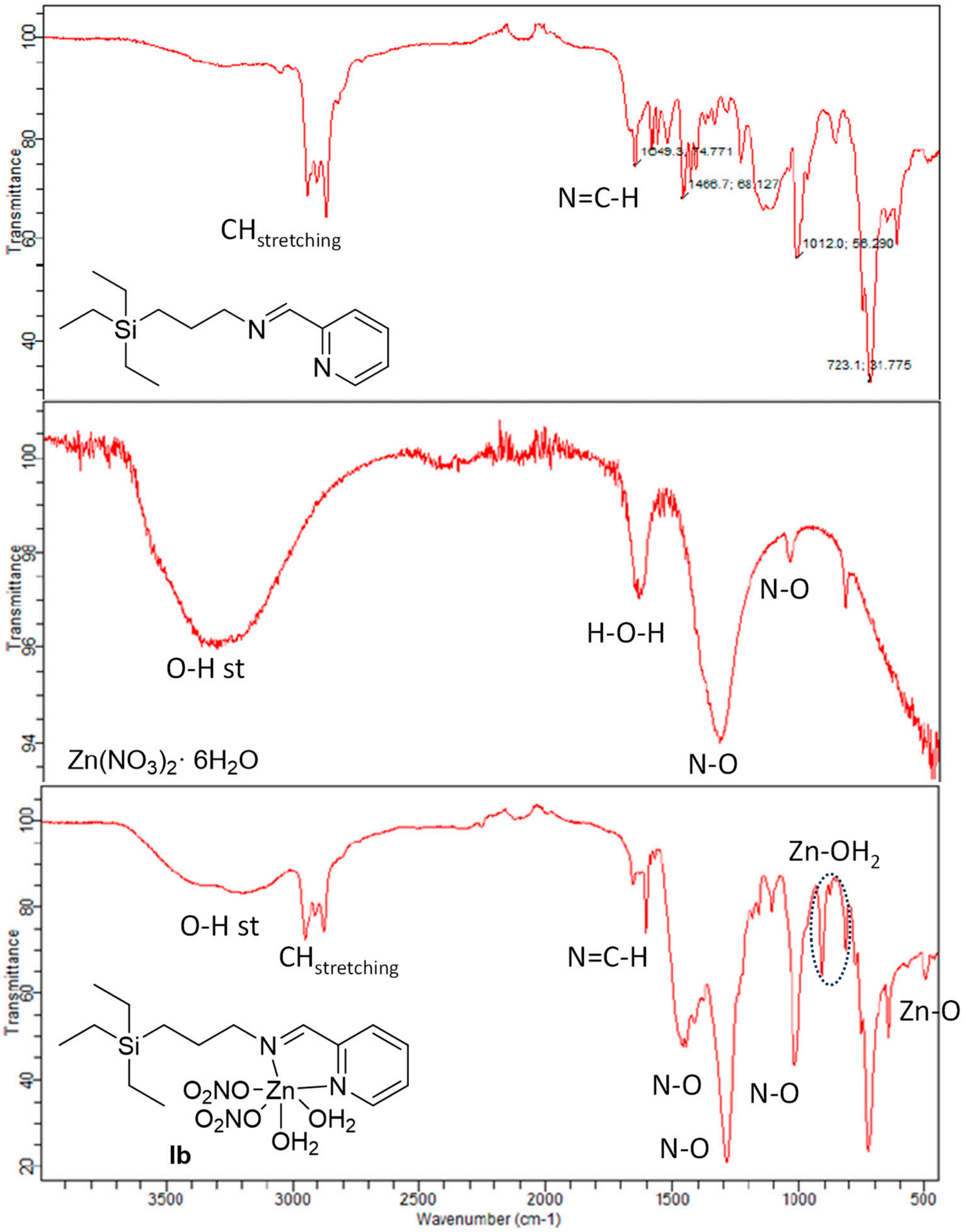

Figure S3. FTIR of compound **I** (top), $Zn(NO_3)_2{\cdot}6H_2O$ (middle) and **Ib** (bottom).

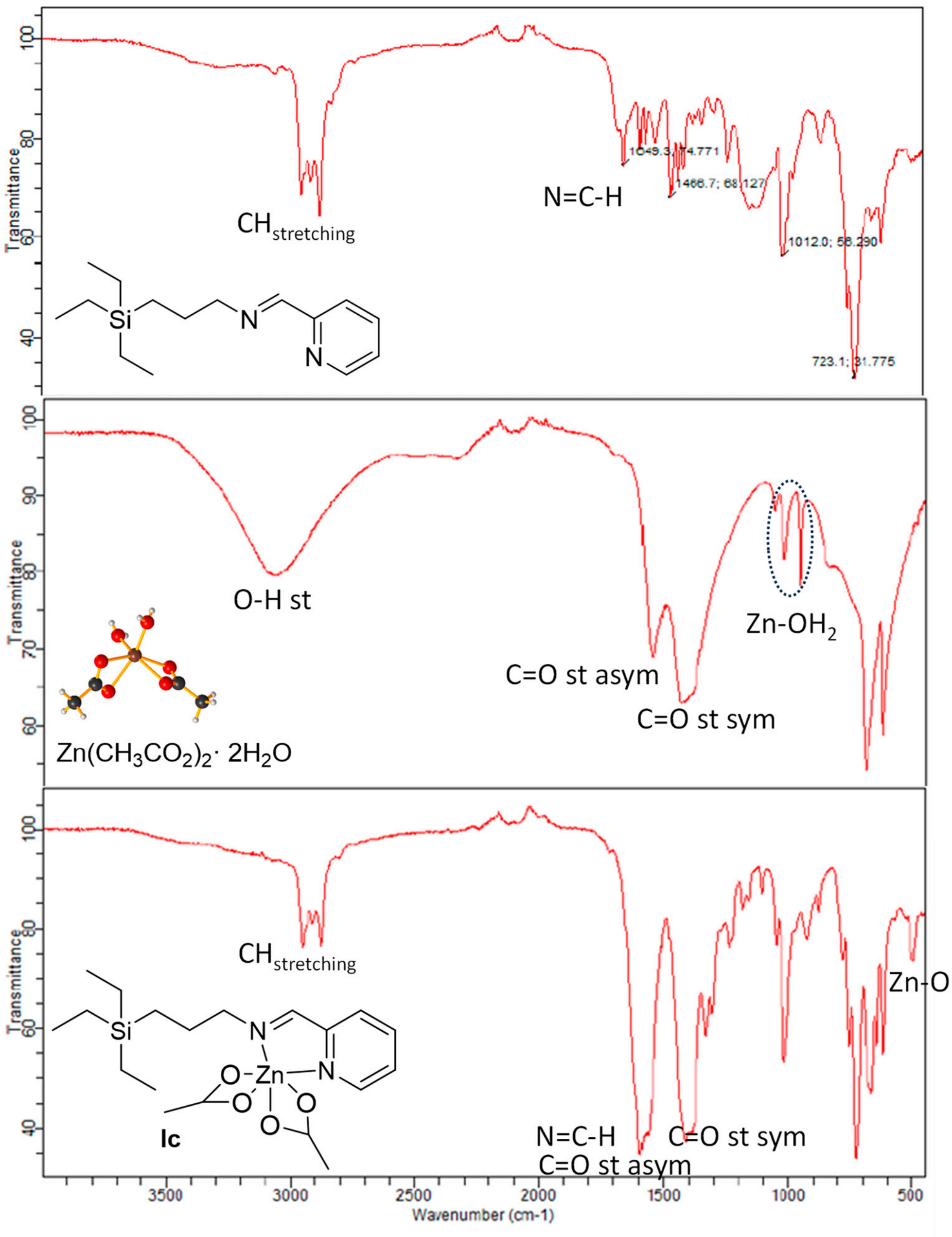

Figure S4. FTIR of compound **I** (top), Zn(acet)$_2$·2H_2O (middle) and **Ic** (bottom).

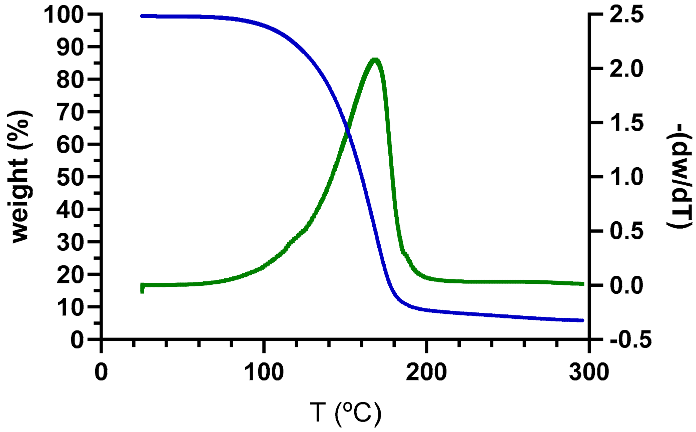

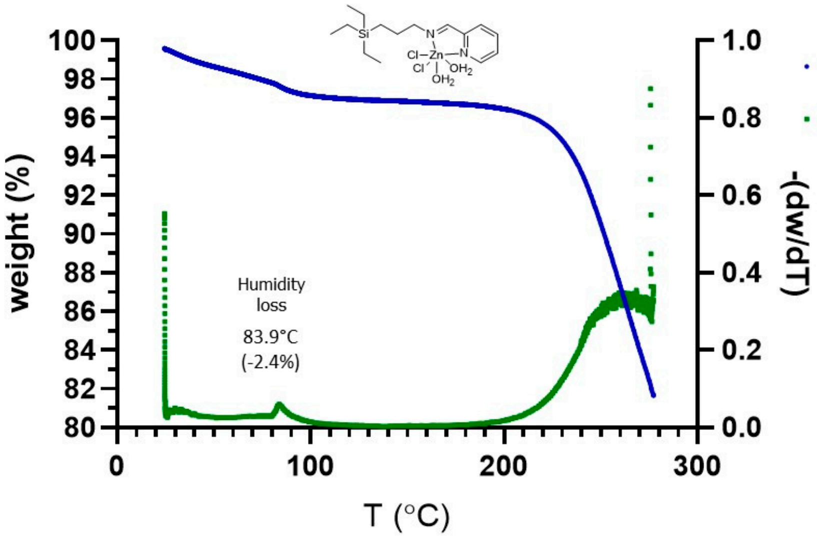

	% loss
**Ia** (Mw 434,78)	
-H_2O (x2)	-4.1% (x2)
-Cl (x2)	-8.1% (x2)
Ligand **I**	-60.4%

Figure S5. Comparative TGA curves of compound **I** (top) and **Ia** (bottom), including table with predicted decomposition steps.

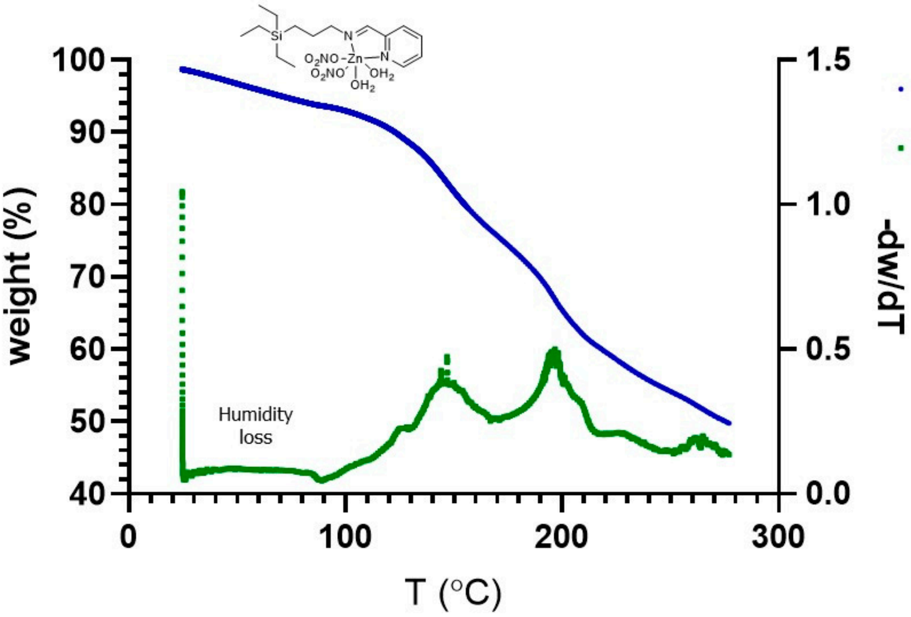

	% loss
**Ib** (Mw 487,89)	
-H_2O (x2)	-3.7% (x2)
-ONO_2 (x2)	-12.7% (x2)
-Ligand I	-53.8%

Figure S6. TGA of compound **Ib**, including table with predicted decomposition steps.

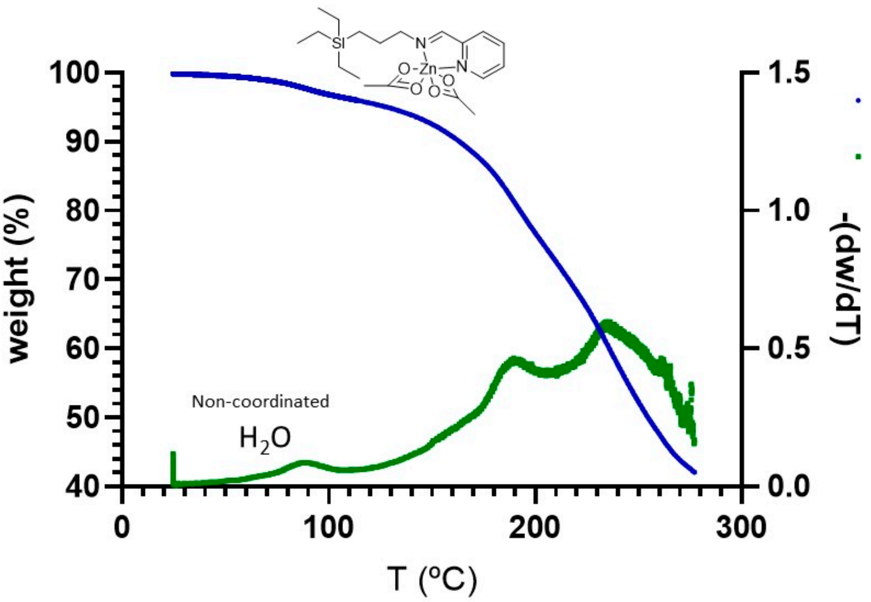

	% loss
Ic (Mw 445,94)	
-acet (x2)	-13,2% (x2)
- Ligand I	-58.9%

Figure S7. TGA of compound **Ic**, including table with predicted decomposition steps.

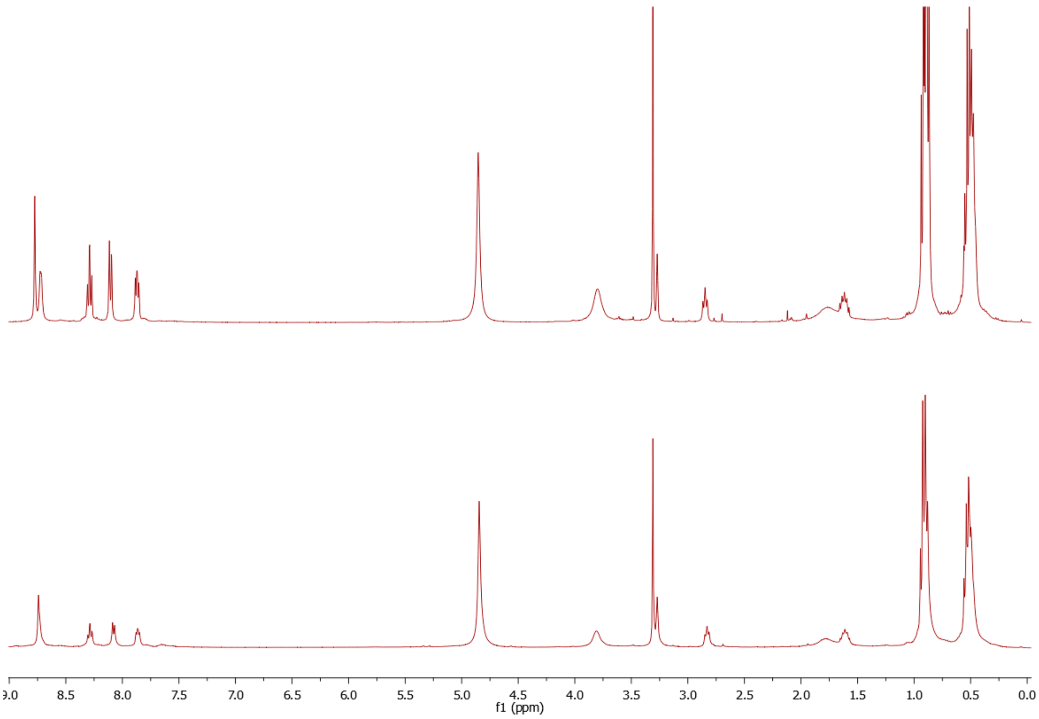

Figure S8. ^{1}H-NMR spectra of compound **Ia** at t=0 (top) and t=7days (bottom) in MeOD.

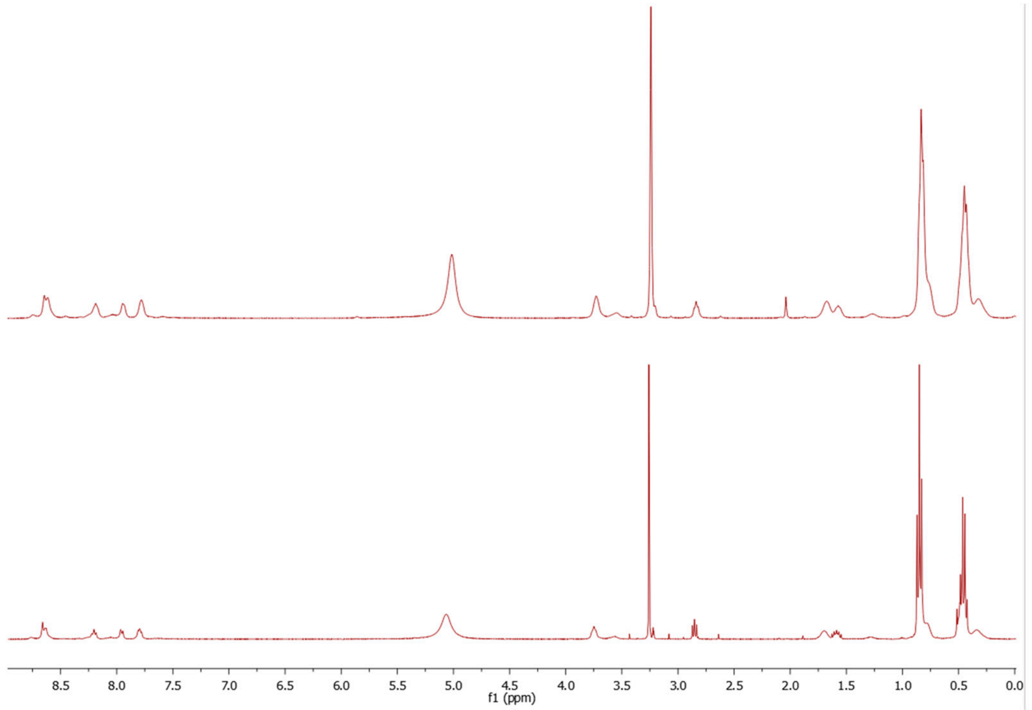

Figure S9. ^{1}H-NMR spectra of compound **Ib** at t=0 (top) and t=7days (bottom) in MeOD.

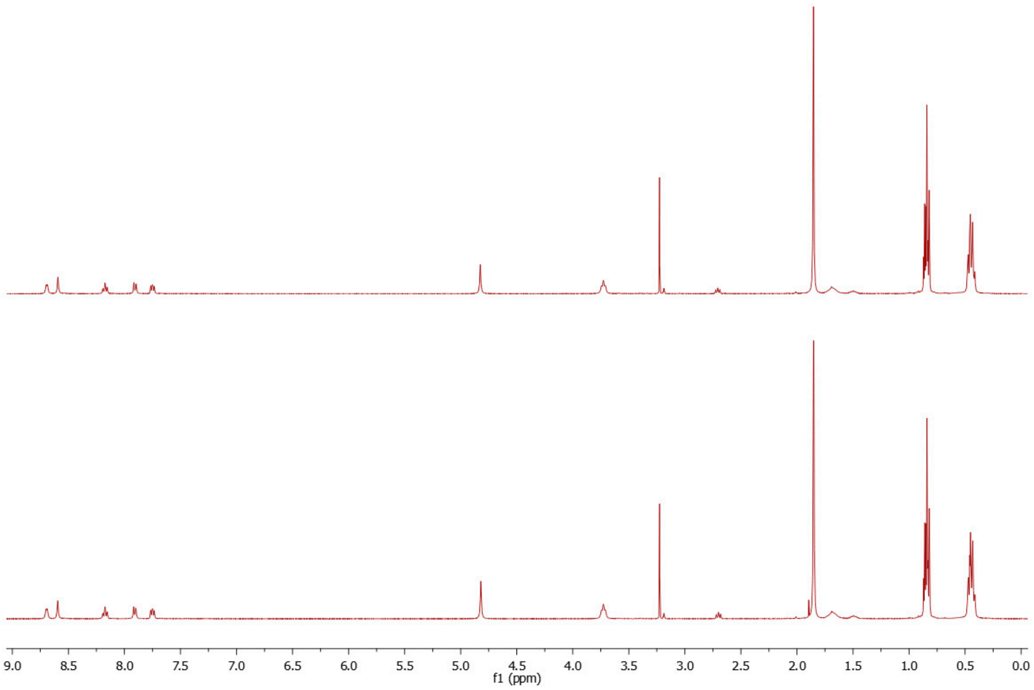

Figure S10. ^{1}H-NMR spectra of compound **Ic** at t=0 (top) and t=7days (bottom) in MeOD.

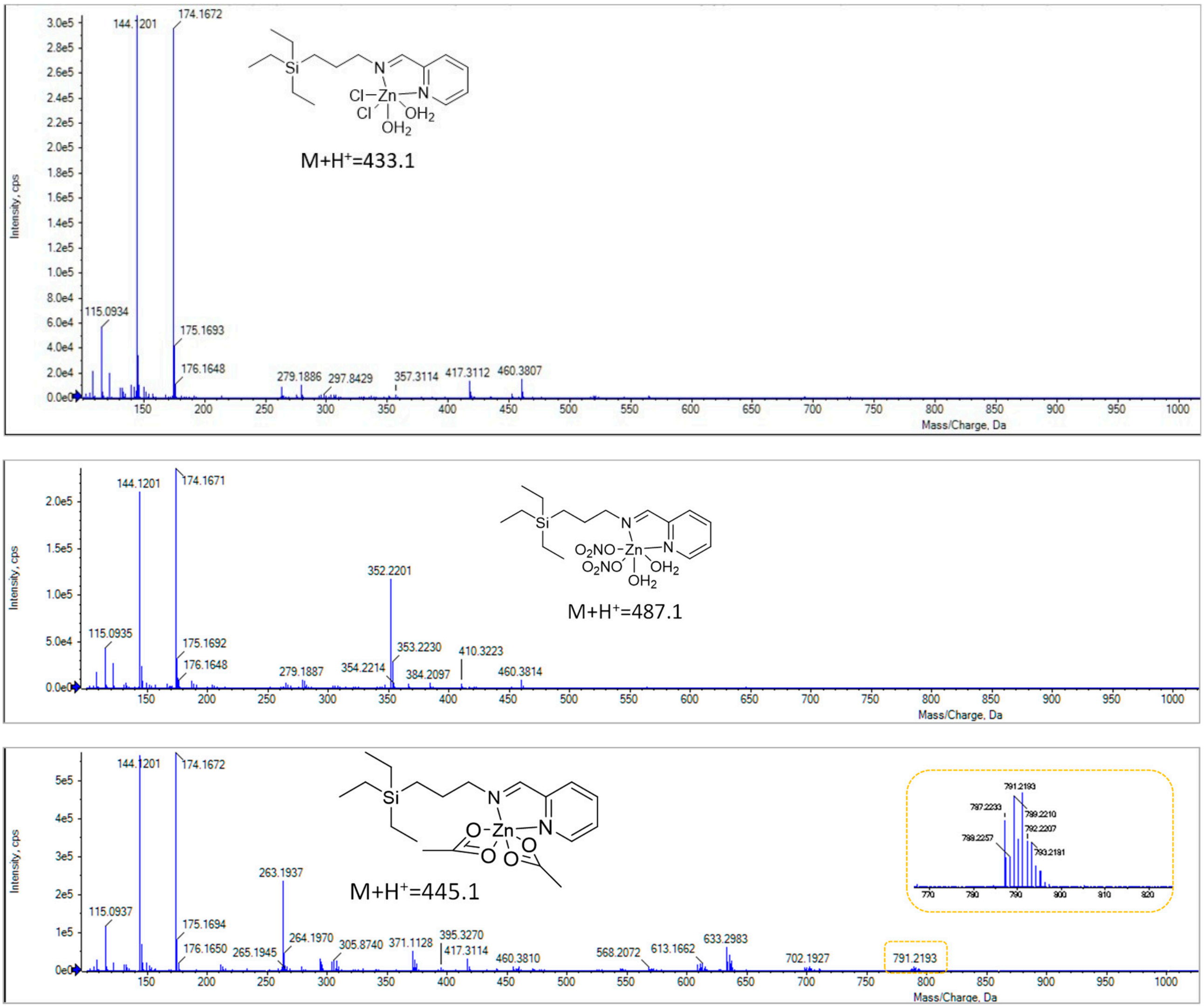

Figure S11. HRMS spectra of compounds **Ia, Ib and Ic**.
